# Expressivism, Relativism, and the Analytic Equivalence Test

**DOI:** 10.3389/fpsyg.2015.01788

**Published:** 2015-11-24

**Authors:** Maria J. Frápolli, Neftalí Villanueva

**Affiliations:** ^1^Department of Philosophy, University College LondonLondon, UK; ^2^Department of Philosophy I, University of GranadaGranada, Spain

**Keywords:** context-dependence, assessment relativism, expressivism, Frege, pragmatism, compositionality, principle of context

## Abstract

The purpose of this paper is to show that, pace (Field, 2009), MacFarlane’s assessment relativism and expressivism should be sharply distinguished. We do so by arguing that relativism and expressivism exemplify two very different approaches to context-dependence. Relativism, on the one hand, shares with other contemporary approaches a bottom–up, *building block*, model, while expressivism is part of a different tradition, one that might include Lewis’ epistemic contextualism and Frege’s content individuation, with which it shares an *organic model* to deal with context-dependence. The building-block model and the organic model, and thus relativism and expressivism, are set apart with the aid of a particular test: only the building-block model is compatible with the idea that there might be analytically equivalent, and yet different, propositions.

## Introduction

MacFarlane (2014, p. 172) has recently claimed that his own kind of relativism and contemporary expressivism, more specifically the one defended by Allan Gibbard, use ‘essentially the same compositional semantics.’ This claim, despite being accurate concerning the semantic value of the specific sentences that McFarlane’s focuses on, might blur a fundamental difference between the expressivist analysis and other semantic approaches. Expressivism, we will argue, is in general compatible with standard compositional semantics, but its basic take on how propositional contents are individuated concedes priority not to the principle of compositionality, but rather to the principle of context. Under expressivism, content is individuated by the inferential import, and thus the compositionalist – building-block – order of explanation is challenged.

The aim of this paper is threefold. First, we will contrast two different models to accommodate context-dependence—the idea that explaining our linguistic practices requires both linguistic and contextual information. The *building-block* model, on the one hand, and the *organic* model, on the other, can be set apart by taking into consideration whether they give prominence to the principle of compositionality over the principle of context, or the other way around. Second, we will argue that expressivism, unlike relativism and other competitors, fits snugly under the latter, organic, model. Third, we will propose a test to determine whether a given theory belongs to the building-block or the organic model – if it is possible for a theory to accommodate the idea that there are analytically equivalent propositions that nevertheless differ, then this theory belongs to the compositional group. According to this test, the *analytic equivalence* test, assessment relativism belongs to the building-block model, while expressivism remains an alternative for advocates of the organic model.

## Context-Dependence: The Building-Block Spectrum

Almost no theory of meaning available aspires to explain our meaningful communicative exchanges in a way that is completely independent from contextual considerations. An elaborate example of this extreme view might be Stojanovic’s *what is said* (Stojanovic, 2007), where content is explicitly designed to be neutral with respect to context-dependent parameters. At one level or another, though, most theories of meaning assume that whatever we can say about the meaning of a string of symbols, as viewed in isolation, differs from what a normal speaker would say while uttering it, or an audience would get while understanding it.

Under the *building-block model*, meaning’s order of explanation proceeds in successive stages, starting from the most basic considerations, and building up from them. At any level, information from the context might be acknowledged by different theoretical alternatives. Here there are some examples. With speech-act pluralism Cappelen and Lepore (2005) claim to put forward ‘insensitive’ semantics, meaning context independent, but they move most contextual effects to the realm of pragmatics, making the communicated information ultimately dependent on the context. Some other “minimalist” alternatives include in the semantic content only the contextual information that is retrieved with the aid of the linguistic meaning of certain expressions, such as indexicals (Stanley, 2000). Sometimes contextual information is meant to have both an impact on what is said as well as on what is globally communicated. Pragmatic explanations of the opacity of belief reports tend to exhibit this feature (see Salmon, 1986, but also Saul, 1998). These theoretical alternatives thus concede a place to contextual information, but are not usually dubbed ‘contextualists,’ because they explain in a context-independent way speakers’ intuitions about the truth of what is said. Contextualists, on the other hand, explain our semantic intuitions by appealing to contextual information.

Within the realm of contextualism, indexical and non-indexical contextualism (cfr. MacFarlane, 2007, 2014) should be distinguished both from Truth-Conditional Contextualism (cfr. Recanati, 2010) and Relevance Theory (Carston, 2002). ‘Indexical contextualism’ is the general label for views according to which the context affects the semantic value of the subsentential linguistic items. Non-indexical contextualism, by contrast, restricts certain contextual processes to the realm of post-semantics. Truth-Conditional Pragmatics and Relevance Theory are instances of “radical contextualism” (Searle, 1992; cfr. Recanati, 2002, p. 303) – whose central motto is that there is no truth-evaluable level of meaning which is unaffected by contextual information.

Assessment relativism (MacFarlane, 2014) recognizes the impact of contextual information on our intuitions about the truth of what we say, but makes it so that some contextual information can be accessible only from a particular context – that of assessment. On occasions, it is not the context in which the sentence is uttered that matters, but the context in which the utterance is received. This type of context-dependence is usually set apart from the aforementioned versions of contextualism, even if it has been argued that the alleged benefits of this view—specially those concerning disagreement—can be accommodated within enhanced contextualist approaches (see, e.g., Kölbel, 2009; Lopez de Sa, 2015, but also Marques and Garcia-Carpintero, 2014; Marques, 2015). At times, it has even been conflated with certain context-dependent approaches (expressivist approaches) whose starting point seems to be quite distant from the building-block model (vid. Field, 2009, p. 252^1^, but cf. Yalcin, 2011, p. 327). We will show in the third section of this paper that assessment relativism truly belongs to the building-block model, and in doing so we will be able to establish a principled difference between this form of context-dependence and another common alternative, i.e., expressivism.

This quick list is by no means intended to be exhaustive; it is meant only to show the spectrum within which different takes on context-dependence can be accommodated. Whether we admit only a minimal amount of contextual information, or we are radical contextualists, we form part of the building-block model if contextual information enters a step-by-step process of meaning construction that starts from the meanings associated with subsentential components, to arrive at a later stage to a complete content.

Depending on the *stage* at which contextual information has an impact, pragmatic processes under the building-block model might be:

Prelinguistic. Input: unsegmented marks or sounds, not recognized as signs belonging to a language. Output: a piece of discourse.

Lexical. Output: a univocal string of words. Take ‘I saw her duck under the table’; only after ‘duck’ is interpreted, either as a verb or a noun, do we proceed to the following stage.

Syntactic. Output: a univocal structure. Compare ‘every ball has a red dot on it,’ and ‘every kid at school has a pet.’ The second sentence exhibits a syntactic ambiguity. Even though a single red dot cannot be on every ball, every kid in the school can be truly said to have a pet if either they are given a different pet for every different kid, or they all treat the school turtle as their very own pet.

Pre-semantic. Output: a univocal set of meanings-cum-structure. Reference fixing for indexicals and semantic disambiguation are commonly assumed to require contextual information.

Semantic. Output: a proposition. Quantifier domain restriction (see Stanley and Szabó, 2000), modulation (see Recanati, 2004, *passim*, see for instance p. 136 and ff.), etc. are typically associated with *local* pragmatic processes.

Post-semantic. Output: a proposition plus a circumstance of evaluation. Typically associated with *global* processes.

Pragmatic. Output: multiple propositions. Secondary inferential processes, for the most part taken to be not sub-personal. Implicatures.

Depending on the *way* in which contextual information is accounted for, pragmatic processes might be:

Primary/secondary. For theories that defend a principled distinction between the semantic core of our utterances and other levels of meaning conveyed, primary pragmatic processes will be those affecting the semantic core, *what is said*, and secondary pragmatic processes will derive other layers of propositional content inferentially from what is said plus other contextual considerations. The latter will typically have an impact at the pragmatic level, even though interactions with other, lower levels are recognized by some approaches, such as Relevance Theory.

Local/global. Local pragmatic processes have an impact on subsentential phrases, global pragmatic processes modify the circumstances of evaluation, placing the whole sentence, as it were, in a different light to be evaluated. These are usually identified at the post-semantic level.

Mandatory/optional. A pragmatic process is mandatory if its intervention is necessary in order to arrive to a level of content that can be evaluated as true or false. Otherwise, it is optional.

Mandatory^∗^/optional^∗^. A pragmatic process is mandatory^∗^ if its intervention is “recruited” by the linguistic meaning of a lexical item, as occurs in the sentence. Otherwise, it is optional^∗^. Indexicals trigger mandatory^∗^ pragmatic processes. These processes are also sometimes deemed ‘bottom–up’ vs. ‘top–down’ processes.

Context-dependence under the building-block model covers the vast majority of theories of meaning in the market. So much so that it is often forgot that there are alternatives to this spectrum of theories. Within this model, contextual information finds its way into the explanation of linguistic communication as part of a progressive building process. But, as we will see in the following section, there are well-known semantic alternatives that exhibit a completely different kind of context-dependence. This form of context-dependence might look alien to some, but it is exemplified by among the best-known theoretical approaches of the analytical tradition. As we will see, the basic insight of this alternative approach was shared by Frege and David Lewis, to mention only two well-known examples. Under the *organic model* context plays a truly preeminent role. Putting context first is what Frege, Lewis, and others did, and it is also part of the agenda put forward by contemporary expressivism^2^.

## Propositional Priority and the Organic Model

In the *organic model*, content individuation is not an issue of assembling pieces into a particular shape. Rather, the basic unit of analysis has to be able to move the chain on the conversational scoreboard, and thus the analysis should take as primitive only linguistic units that can be used to acquire certain inferential commitments. Context is not needed to fill in the holes left in the logical form by semantic underdetermination, but rather to supply the information that is needed to make sense of a certain communicative exchange.

Dealing with contextual information organically requires being able to apply the contribution of the context to the content expressed, and this in a way that cannot be specified by taking into account how the linguistic meaning of the subsentential bits becomes modified when introduced in that particular situation, only to be afterward assembled in a meaningful whole. As we saw in the previous section, whether we take the contextual information to be gathered with the aid of linguistic instructions – through mandatory^∗^ pragmatic processes, or freely – as the result of optional^∗^ processes, or secondary pragmatic processes, the building-block model would always proceed from subsentential units to a whole proposition. The organic model needs to start from a completely different stance. No longer would it suffice to check how the contextual information bears upon the particular meaning of the phrases as they are currently used, a large amount of contextual information can also have an impact on the content which cannot be domesticated into the modulation of some pieces of the whole. The starting point of the organic model is the content of judgments, whatever we can put forward as a premise or a conclusion, what we stand for and become responsible for in a conversation.

In communicative acts the immediate data are contents of propositional nature, expressed by sentences. These contents are individuated within a given context, and this makes the organic model context-dependent, even though context provides information in a way that cannot be equated to those mentioned above. To “move the chain,” agents have to perform some kind of act, since acts are the minimal moves in the communicative game. Brandom gave flesh to this classical pragmatist intuition: ‘sentences are the kind of expression whose freestanding utterance […] has the pragmatic significance of performing a speech act’ (Brandom, 2001, p. 125). ‘Without expressions of this category,’ Brandom went on, ‘there can be no speech acts of any kind, and hence no specifically linguistic practice’ (loc.cit). Both logically and chronologically, rational agents’ first contact with language is somebody saying something. Only afterward is the identification of words and structures available.

Lewis’ epistemic contextualism (Lewis, 1996) is a well-known example of an organic use of contextual information. His view cannot be forced into any of the building-block varieties of context-dependence introduced in the first section of the present paper. Lewis faces the challenge of the skeptic, and provides a definition of knowledge that can, on the one hand, explain why the skeptic maneuver makes sense, as traditionally discussed in epistemology, and, on the other hand, the fact that we truly know many things. The skeptic, by continuously forcing us to look at alternatives that we had not previously considered, makes us doubt our firmest beliefs, and therefore it seems that none of our beliefs can ever after be secured, so as to be called ‘knowledge.’ Lewis’s strategy allows for our knowledge attributions to be true before meeting the skeptic, while our post-skeptic knowledge attributions become false. Meeting the skeptic has exercised a crucial change in the context, and knowledge attributions become context sensitive.

Here is Lewis’ definition:

S knows that P iff P holds in every possibility left uneliminated by S’s evidence —Psst!— except for those possibilities that we are properly ignoring (Lewis, 1996, p. 561).

‘S knows that p’ will then be true if every alternative in which not p is eliminated by S’s evidence. I know that my pen is inside my bag at 00:35 because I can rule out every possible chain of events leading up to my pen being elsewhere. I saw it in the bag a minute ago, lighting conditions are ok, I am under the influence of no perception-altering substances, nobody has entered the room since the last time I saw it in the bag, etc. My evidence eliminates every possibility in which my pen is not in my bag. If I know it, my attribution at 00:35 will always be true. But, ‘is that so?’ the skeptic would ask at 00:36, only to introduce subsequently an exotic alternative, previously ignored, in which my pen is absent from my bag, an alternative that my current evidence cannot eliminate. What if everything I see is nothing but a cleverly produced illusion, conducted by a demon who, as a matter of fact, happens to have my pen in his hand? I can no longer truly say that I know that my pen is in my bag, since my evidence tells me nothing about the existence of that demon. How can my attributions differ so drastically in a minute? Lewis’s response is that the clever skeptic makes it inappropriate to ignore certain possibilities. It was true at 00:35 that I knew that my pen was in the bag, and it is also true that I do not know at 00:36 that my pen is in the bag. Being sometimes susceptible to the reasons of the skeptic does not make me an illogical person.

The context alters the content of the epistemic attribution by changing the alternatives that can properly be ignored, and so it does in a tacit way (thus, Lewis’s ‘psst’). Crucially, the context does not modify any of the subsentential items of the sentence, to make it fit into the conversational occasion. Lewis’s context-dependence of knowledge attributions can be accommodated only within the organic model, one in which we start by looking at the conditions under which a particular judgment, my knowledge attribution in this case, makes sense.

Frege, one of the founding figures of semantic analysis, and therefore an unavoidable reference for current alternatives within the philosophy of language, also assumed the organic model of individuation as the backbone of his logic and semantic proposal. It would be a disservice to restrict Frege’s *organic inclinations* to his first works. Not only did he maintain them in his first significant works, but he also took sides with the principle of context until the end of his career.

The project of defining the concept ‘number’ in ‘Grundlagen’ is an illustration of the organic procedure. Frege exposed the flaws of the classical strategy of defining numbers by putting ‘units’ together and shifted to a different method: ‘It should throw some light on the matter to consider number in the context of a judgment which brings its basic use’ (Frege, 1884/1960, §46, p. 59). This is an application of the second principle that he introduced in the prolog of this work and that defined his logico-semantic project, ‘never to ask for the meaning of a word in isolation, but only in the context of a proposition’ (p. xxii), the principle of context that shaped the development of logic and semantics ever since.

The principle of context is a rich indication that can be understood as making a point about the contextually modulated meaning of the words in a sentence, or else as a statement about the logical priority of propositions over concepts. The first reading, which has become the centerpiece of several varieties of contextualism, elaborates it in the notion of modulation of meaning (vid. Recanati, 2004, p. 39 and ff.). It is nevertheless the second reading that characterizes the organic model. To avoid misunderstandings, we will call this second reading the Principle of Propositional Priority: [Principle of Propositional Priority] Propositions are the primary bearers of logical, semantic, and pragmatic properties.

Two judgments, Frege explains (Frege, 1879, §3), can differ in two ways: (i) From the two of them together with a certain set of premises, the same set of consequences follows. (ii) Alternatively, the sets of their consequences might not coincide. In the first case, the two judgments have the same content; in the second case, their contents are different. A propositional content, the content of a possible judgment, is thus individuated by the contents that follow from it (together with some auxiliary information). In this model, subsentential and subpropositional elements play no essential role in content individuation. As Frege put it:

Let us assume that the circumstance that hydrogen is lighter than carbon dioxide is expressed in our formula language, we can then replace the sign for hydrogen by the sign for oxygen or that for nitrogen. This changes the meaning in such a way that ‘oxygen’ or ‘nitrogen’ enters into the relations in which ‘hydrogen’ stood before. If we imagine that an expression can thus be altered, it decomposes into a stable component, representing the totality of relations, and the sign, regarded as replaceable by others, that denotes the object standing in these relations. The former component I call a function, the latter its argument. *The distinction has nothing to do with the conceptual content; it comes about only because we view the expression in a particular way* (our italics; Frege, 1879, p. 22).

Frege’s approach to the other classical principle, the Principle of Compositionality, is patent in this text. The interpretation of the principle that characterizes the building block model takes it as a criterion of propositional individuation in which propositions are complex entities made up of simpler parts. We claim, nevertheless, that this is not Frege’s interpretation. The organic model is compatible with a view of compositionality as a method of propositional analysis, not as a criterion of propositional individuation. A single proposition can be expressed by different sentences, which open up diverse possibilities of propositional analysis. Even if propositions are, in the organic model, non-structured entities, the structure of sentences can be projected, for the sake of a particular analytic aim, onto the propositional contents expressed by them. This fact should not make us forget that there is a sharp distinction between the ontological characterization of propositions as structured entities build up on blocks, on the one hand, and the semantic project of assigning semantic values to expressions in a sentence, on the other. Lewis (1980)^3^ is an example of the defense of the organic model of propositional individuation and the compositional approach to the semantic value of expressions.

That the classical building-block interpretation of compositionality is alien to Frege’s thought is no news any more. It has been defended by (Jansen, 2001) and (Pelletier, 2001), among others. In what follows we will offer new evidences^4^.

From the principle of propositional priority follows one of Frege’s longstanding insights, one that plays a particularly relevant role in this paper and that is the core of the organic model: it makes no sense to admit the possibility that there might be different, yet analytically equivalent, thoughts, an insight, we contend, that is not compatible with the building-block model. Furthermore, as a defining feature of the organic approach to propositions, it serves as a test to set apart two essentially distinct uses of context, as we will do in the next section of this paper. If propositional contents are organically individuated, analytically equivalent sentences express the same proposition. This claim amounts to a rejection of a possible isomorphism between sentences and the propositions expressed by them, and is a major consequence of the organic model. In Frege’s writings the rejection of the isomorphism between sentences and thoughts is represented by his move toward contents by overlooking the grammatical surface of judgments. Languages serve thoughts to get ‘clothed in the perceptible garb of a sentence’ (Frege, 1918–1919a, p. 354) and can be used ‘as a bridge from the perceptible to the imperceptible’ (Frege, 1923–1926, p. 259). Nevertheless, cloth and flesh, the perceptible and the imperceptible maintain their independence, and the principle of propositional priority establishes which one takes the lead. In ‘Logical Generality’ (Frege, 1923–1926), for instance, Frege says: ‘We should not overlook the deep gulf that yet separates the level of language from that of the thought, and which imposes certain limits on the mutual correspondence of the two levels’ (Frege, 1923, p. 259). Passive transformation becomes one of his favorite examples. From ‘Begriffsschrift’ to ‘Logical Investigations,’ he resorts to it to show that non-synonymous sentences (in the standard sense) can systematically be used to elicit the same thought:

A sentence can be transformed by changing the verb from active to passive and at the same time making the accusative into the subject. In the same way we may change the dative into the nominative and at the same time replace ‘give’ with ‘receive.’ Naturally such transformations are not trivial in every respect; but they do not touch the thought, they do not touch what is true or false (Frege, 1918–1919a, p. 357).

But passivization is not the only case. Frege’s substitution mechanism to determine the contribution of subsentential expressions is a further example of his use of context, a mechanism that Brandom takes over to explain the inferential function of singular terms and predicates (Brandom, 2001, Chap. 4 *passim*). ‘Frege was the first,’ Brandom concedes, ‘to use distinctions such as these to characterize the roles of singular terms and predicates. Frege’s idea is that predicates are the substitutional sentence frames formed when singular terms are substituted for in sentences’ (Brandom, 2001, p. 131).

An example of a different sort that nevertheless illustrates the same point occurs in the realm of logic. Logical terms mean unsaturated notions whose arguments can be sentences, truth-values or thoughts, depending on the perspective we take on them. In particular, thoughts can be compounded to form more complex ones by means of logical operations. Nevertheless, the logical operations applied to sets of thoughts can be rendered in natural and logical languages through sentences with different ingredients. The thought expressed by any instance of the schema ‘(A & A)’ is the thought expressed by the corresponding instance of ‘A’ (Frege, 1923–1926, p. 393, n. 21). The thought expressed by any instance of the schema ‘Not [(not A) and (not B)],’ is the thought expressed by the corresponding instances of ‘Not [Neither A not B]’ and by the corresponding instances of ‘A or B’ (Frege, 1923–1926, p. 396).

Thus, even if Frege explicitly uses the building-block image (as in Frege, 1914, p. 225), he takes the idea that thoughts are made out of simpler parts that correspond to the parts of the sentences metaphorically. In ‘On Sense and Meaning’ he says:

Here, I have used the word ‘part’ in a special sense. I have in fact transferred the relation between the parts and the whole of the sentence to its meaning, by calling the meaning of a word part of the meaning of the sentence, if the word itself is a part of the sentence. This way of speaking can certainly be attacked, because the total meaning and one part of it do not suffice to determine the remainder, and because the word ‘part’ is already used of bodies in another sense. A special term would need to be invented (Frege, 1892, p. 165).

At the end of his life, Frege still maintains the same view:

If one thought contradicts another, then from a sentence whose sense is the one it is easy to construct a sentence expressing the other. Consequently the thought that contradicts another thought appears as made up of that thought and negation […]. But the words ‘made up of,’ ‘consists of,’ ‘component,’ ‘part’ may lead to our looking at it the wrong way. If we choose to speak of parts in this connection, all the same these parts are not mutually independent in the way that we are elsewhere used to find when we have parts of a whole’ (Frege, 1918–1919b, p. 386).

In summary, the Fregean principle of propositional priority introduces a way of individuating propositional contents that makes an idiosyncratic use of context, a use that cannot be accommodated in any of the contemporary positions that attempt to harbor the effect of contextual factors in what is said. The Fregean organic model and the compositionalist model that serves as a background for the theories depicted in the first section of this paper stand in sharp contrast with profound philosophical consequences^5^. The two models are incompatible, as we will show in the next section.

## Expressivism and the Organic Model

It is the purpose of this section to show that contemporary expressivism, at least in the way in which some of its most popular varieties are commonly understood, is incompatible with the building-block model. We do so by focusing on the expressivist’s commitment with the idea, mentioned in the previous section, that there cannot be different, and yet analytically equivalent, propositions. In so doing, we will argue for a somewhat controversial statement (see, for example Field, 2009, p. 252) that we introduced in the first section of the paper – that MacFarlane’s assessment relativism, as a representative of the building-block model, needs to be sharply distinguished from expressivism, a paradigmatic example of the organic model.

Classical Expressivism analyzes sentences with ethical terms, such as ‘cheating on your husband is bad,’ as having the general import of ‘boo for cheating!,’ i.e., as interjections devoid of propositional content that cannot qualify as true or false. Contemporary expressivism, by contrast, acknowledges an evaluable content, organically individuated, to acts with expressive terms. The ‘expressivist’ strategy,’ as Gibbard puts it ‘is to change the question. Don’t ask directly how to define ‘good’… shift the question to focus on judgments: ask, say, what judging that is good consists in’ (Gibbard, 2003, p. 6). This pattern applies to a wide variety of topics. Gibbard (2012) applies it to semantics, Chrisman and Field to knowledge ascriptions (Chrisman, 2007, 2012; Field, 2009; Carter and Chrisman, 2012), Bar-On to first-person ascriptions (Bar-On, 2004), and so on.

In an expressivist setting, the content of normative claims is individuated organically. Higher-order functions, functions like ‘is wrong,’ ‘is good,’ ‘S knows that,’ ‘S believes that,’ or ‘necessarily,’ are non-truth-conditional functions that do not describe how the world is. Some of these functions are functions of propositions whose semantic role does not consist in adding a conceptual component to the propositional content of the communicative act in which they are used. For those, expressivists like Gibbard (2012, p. 179) propose an *oblique* approach – by focusing on the mental states that are expressed by the use of normative utterances, inferential relations of entailment and incompatibility are exposed, and these are the touchstone of the expressivist analysis.

So far, the characterization of the meaning of functions of propositions is negative: they are non-truth-conditional, non-descriptive, non-contributive. But if they do not describe the world and do not contribute to the proposition, what semantic role do they perform? How are they individuated? A temptation for many expressivists, old and new, is to identify the meaning of the relevant terms with some kind of mental state, attitude or feeling. An example is Gibbard (1990): ‘According to any expressivistic analysis, to call something rational is not, in the strict sense, to attribute a property to it. It is to do something else: to express a state of mind’ (Gibbard, 1990, p. 9). But, as we argued in (Frápolli and Villanueva, 2012, p. 485), this is unnecessary, ‘since the meaning of these expressions is exhausted once their inferential potential is indicated.’ A look at this inferential potential makes it apparent that normative expressions are distinctively connected with other expressions that include functions of propositions – they entail some, they are incompatible with some others, and that these connections suffice to explain their semantico-pragmatic behavior. In the next few paragraphs, we sketch the kind of minimal expressivist analysis of functions of propositions that we have developed in Frápolli and Villanueva (2012).

To give the meaning of ‘S believes that p’ – we consider modal, but also doxastic and epistemic, attributions to belong to the realm of the normative – is to identify the circumstances under which an agent is entitled to utter this sentence, and the consequences that can be derived from the attribution. It is constitutive of the meaning of ‘believe’ that an agent cannot attribute to a subject the belief that p and at the same time the belief that p cannot be true. This is the standard truth norm. Attributing beliefs to an agent commits the attributor to the further attribution of plans to act according to his/her beliefs. If we attribute to Victoria the belief that she is late for work, we should attribute to her the intention to leave immediately (even if factors preclude her from acting in this way).

Similarly, the meaning of ‘know’ is such that if an agent attributes to a subject the knowledge that p, the agent will be committed to the truth of p. Attributing the knowledge of p is incompatible with our belief that p is false. As M. Williams puts it, ‘in attributing knowledge to another person, I concede both the truth of what he believes and his right to believe it. And in advancing this double endorsement, I take on the same commitments and lay claim to the same entitlements’ (Williams, 2001, p. 17). Knowledge and belief are different concepts *because* the conditions for their use and the commitments acquired by their attribution do not coincide.

The same can be said of pairs of logical terms such as ‘or’ and ‘and.’ Utterances of ‘Yum likes licorice and Yuk dislikes it’ and ‘Yum likes licorice or Yuk dislikes it’ express different contents because the latter, unlike the former one, is compatible with the assertion that Yum dislikes licorice. Conjunction and disjunction are distinct concepts *because* they derive from, and produce, distinct permissions and prohibitions, i.e., because they sanction divergent behavioral responses. Generality and instantiation likewise give rise to different permissions and commitments. A rational agent cannot believe that an individual *x* has the property *P* and at the same time reject that something is *P*, for *Px* and *It is not the case that there is a P* express incompatible contents. On the other hand, if two expressions systematically give rise to the same set of commitments and share the circumstances under which they can be properly used, they also share their content. One might feel that the meanings of ‘every’ and ‘all’ are slightly different. In fact, they are not universally interchangeable *salva congruitate*. But if there is no detectable difference in claiming that *every child likes football* and *all children like football* in terms of the agent’s entitlements and commitments, there is just one proposition expressed by the two claims. And the same happens with the following sentences,

‘tame tigers exist,’
‘some tigers are tame,’
‘there are tigers that are tame,’ and
‘not all tigers are not tame.’

These sentences are not isomorphically identical; some words occur in some of them and not in others, and they do not possess the same structure. But the inferential moves that would be made by the use of any of them in a communicative act would not be affected by the replacement of any one by any of the others, for nothing follows from any of them that does not follow from the others too.

Now it should be patent that the expressivist approach hinted at so far falls within the organic model. The content of the set of expressions to which the expressivist analysis applies is individuated by reference to the inferential links granted or precluded by assertions in which they occur, rather than by factoring in the modulated meanings of subsentential items. This minimal brand of expressivism we take to be compatible with the core of major contemporary expressivist approaches, and the features that make this position an example of the organic model belong to this common core^6^.

Our claim about the organic nature of the expressivist enterprise will be now assessed with the aid of the test that we introduced in the previous sections. Expressivism is naturally committed to the idea that there cannot be different, but analytically equivalent, propositions, and this tenet has been put to use as a premise by John MacFarlane in an argument to undermine the expressivist analysis of predicates of personal taste. We will show how, even though he is right in attributing that principle to the expressivist, his argument does not work. In the process, the crucial difference between expressivism and relativism will become apparent.

John MacFarlane has, in recent years, developed an analysis of predicates of personal taste that makes them context-dependent but that also differs from most previously known versions of contextualism. According to MacFarlane, what makes these predicates special is that they require the intervention, at a post-semantic level, of certain information that can be gathered only from the context of assessment – rather than the context of utterance. Even if it was true when originally produced, I can retract my claim that ‘licorice is not tasty’ because I am assessing it now in a different context. With this, MacFarlane manages to offer an alternative both to objectivism – the idea that claims that contain predicates of personal taste are true or false *simpliciter* – as well as to contextualism – the idea that every taste claim involves a reference to a set of taste standards. Expressivism, MacFarlane posits, can nevertheless be developed into a position on the matter that looks dangerously close to his own assessment relativism. Not so close, though, that it cannot be differentiated from it, and evaluated accordingly like a different theory.

Relativism and expressivism would offer similar treatments of descriptive beliefs, which are individuated in terms of compatibility and incompatibility among mental states (MacFarlane, 2014, p. 170). The conflict would arise when assessment-sensitive beliefs are considered. In these cases, MacFarlane’s reconstruction of the expressivist position would offer an indirect characterization of beliefs via the language of preference. In such an expressivist framework, to attribute to someone the belief that licorice is tasty would be to attribute to that individual ‘the very same kind of state’ (MacFarlane, 2014, p. 173) that we would make the attribution by saying that he/she likes licorice. By contrast, McFarlane’s relativism rejects the contention that beliefs with taste-relative contents can be identified with any ‘state we could attribute using the language of preference’ (ibid.). ‘Why might it matter whether there is one state or two?’ he asks. And his answer brings into the open a qualitative difference between the two accounts: for an expressivist it is ‘conceptually impossible to think that something whose taste one knows first-hand is tasty while not liking its taste.’ This, MacFarlane argues, would be going too far:

The relativist […] can agree that the questions are ‘not separate’ in the following sense: first-person deliberation about each gets resolved by the same considerations. It does not follow from this, however, that the questions concern the same psychological state (MacFarlane, 2014, p. 174).

In other words, a first-person avowal of a belief with the content that licorice is tasty is practically indistinguishable from a claim to the effect that the speaker likes its taste. But even so, an agent who is working hard to improve his/her taste standards could make sense of a situation in which he/she still likes licorice but would be willing to accept that it might not be tasty after all. And clearly, a subject who assesses these avowals can mark the first one as saying something false while ascribing truth to the second claim.

Thinking that we like the taste of something having a taste we know first-hand, and thinking that something is tasty are conceptually, i.e., analytically, equivalent, and yet, MacFarlane argues, an agent can be in a position in which it is not irrational to attribute one but not the other. Only MacFarlane’s own assessment relativism can account for this fact. Expressivism, no matter how close to relativism it might appear, is necessarily committed to the opposite idea. McFarlane’s remarks disclose an irresoluble conflict with expressivism that can be expressed in terms of the two models of content-individuation described in the foregoing sections of the present paper. If McFarlane’s diagnosis is accurate, relativism and expressivism come apart at this deeper level, and, to the despair of the proponents of the organic model, there is something intuitively correct in the idea that one might acknowledge that something is tasty without liking its taste herself.

Thus, once it is assumed that the building-block model and the organic model can be used to spell out the differences between such close views as assessment relativism and expressivism, MacFarlane’s arguments could be taken even a step further, to use them against the whole organic model. If ‘Licorice is tasty’ and ‘I know the taste of licorice first-hand and I do like it’ are analytically equivalent, but it can be rational to believe one but not the other, maybe it is inappropriate as a general policy to claim that there cannot be different but analytically equivalent propositions. We close this paper by providing some evidence in favor of the organic take, arguing (i) that ‘Licorice is tasty’ and ‘I know the taste of licorice first-hand and I do like it’ are not analytically equivalent within the organic model, and (ii) that, with respect to those sentences that are declared to be analytically equivalent by the organic model, it is indeed irrational to believe one but not the other. A crucial aspect of our argument is the rejection, already mentioned, of the idea that the job of sentences like ‘Licorice is tasty’ and ‘I know the taste of licorice first-hand and I do like it’ is to voice mental states. We are willing to accept, as both McFarlane and Gibbard do, that the state of mind that makes an agent utter any of them might be identical. But it does not follow from this that they are analytically equivalent. Their meanings do not equate to the expression of feelings or attitudes but they are instead individuated by the inferential commitments that a speaker acquires when uttering them, commitments that belong to the public sphere and for which what happens in the agent’s head is strictly irrelevant.

The general question underlying the conflict does not have an easy answer. Whether it makes sense to accept that two contents can be different even if the sentences by means of which we systematically express them are analytically equivalent crucially depends on the kinds of concepts involved. For ordinary first-level sentences, i.e., the kinds of sentences that express what Ramsey calls beliefs of the ‘primary sort’ (Ramsey, 1929, p. 146) and Boole and Frege ‘primary propositions’ (Frege, 1880–1881, p. 14), the possibility of finding cases of the kind put forward by Benson Mates (Mates, 1952), always seems open. Under the organic model, as we examined in Section “Propositional Priority and the Organic Model,” content is individuated inferentially, but that does not mean that no mechanism can be devised to check whether or not two particular linguistic items, sentential or subsentential, express the same content. Whenever two expressions are not interchangeable *salva veritate*, it is proved that their inferential behavior crucially differ, and therefore cannot be taken to express the same content. Mates’ cases are a particularly telling way to explore the inferential content of pairs of expressions.

(1a) Nobody doubts that, whoever believes that ophthalmologists are ophthalmologists, believes that ophthalmologists are ophthalmologists.
(1b) Nobody doubts that, whoever believes that ophthalmologists are ophthalmologists, believes that ophthalmologists are oculists.
(2a) Nobody doubts that, whoever believes that licorice is tasty, believes that licorice is tasty.
(2b) Nobody doubts that, whoever believes that licorice is tasty, believes that he/she knows the taste of licorice first-hand and likes it.

While it still makes sense to ask whether 1b could be false, the truth of 1a goes unquestioned. ‘Being an ophthalmologist’ and ‘being an oculist’ are not, as a consequence, analytically equivalent. Comparing 2a and 2b offers a similar result. While 2a is obviously true, the truth of 2b can be challenged. A person attending a wine-testing course might be rational to think that he/she likes the taste of a certain wine while not thinking that it is tasty. This would not show that two analytically equivalent sentences can be rationally entertained as different, and therefore that expressivism fails. Rather, this would only show that expressivism – or MacFarlane’s reconstruction of it as applied to taste predicates – had gone too far in claiming that those two thoughts are analytically equivalent. Within an expressivist-organic approach it makes perfect sense to think that licorice is not tasty, while still liking its taste, because ‘Licorice is tasty’ and ‘I know the taste of licorice first-hand and I do like it’ are *not* analytically equivalent. Thus 2a and 2b prove that they have different inferential import. MacFarlane’s insistence on ‘Licorice is tasty’ and ‘I know the taste of licorice first-hand and I do like it’ being analytically equivalent, in spite of their distinct inferential behavior, only confirms that his assessment relativism belongs to the building-block model. The expressivist does not need to shy away from MacFarlane’s argument precisely because organic content individuation is incompatible with the claim that ‘Licorice is tasty’ and ‘I know the taste of licorice first-hand and I do like it’ have the same content – they are not analytically equivalent. In fact, MacFarlane’s claim that they are is only possible if relativism falls outside the sphere of the organic model.

‘Being an ophthalmologist,’ like ‘being tasty,’ are first-order predicates. Expressivism, we have claimed, is nevertheless essentially concerned with functions of propositions. Modal, epistemic, doxastic operators, along with ethical terms and logical constants were among the examples that we offered to characterize the view as an instantiation of the organic model. In fact, Frege’s examples, the ones that we introduced in order to argue for the idea that an organic individuation of content was incompatible with the existence of analytically equivalent, and yet different, propositions, involved a difference only in functions of propositions – logical constants, or the operation of passivization. Using the Mates test to check on sentences differing only at the level of functions of propositions proves to offer striking results:

(3a) Nobody doubts that, whoever believes that p, believes that p.
(3b) Nobody doubts that, whoever believes that p, believes that p.
(4a) Nobody doubts that, whoever believes that lawyers are wealthy, believes that lawyers are wealthy.
(4b) Nobody doubts that, whoever believes that lawyers are wealthy, believes that it is not the case that lawyers are not wealthy.
(4c) Nobody doubts that, whoever believes that lawyers are wealthy and researchers are poor, believes that it is not the case that lawyers are not wealthy or that researchers are not poor.

Sentences 3a and 4a are trivially true. But, contrary to what happens with 1b and 2b, sentences 3b, 4b, and 4c also appear to be unquestionably true. A rational agent cannot attribute the belief that lawyers are wealthy without attributing the belief that it is not the case that lawyers are not wealthy. Otherwise the agent would display serious rationality flaws. Those who reject 4c remove themselves from the community of rational agents. In these cases, what is in question is not lexical mastery but the basic understanding of the rules of language.

Concerning functions of propositions, our intuitions agree with the predictions of the organic model. Rational agents cannot believe *that Alan is an expressivist*, without believing *that it is true that Alan is an expressivist*, for what is at stake is a single belief not two beliefs tightly connected. The same content is expressed by the sentences ‘it is not the case that Alan is not an expressivist,’ and ‘Alan is an expressivist or Alan is an expressivist.’

## Conclusion

MacFarlane’s assessment relativism is necessarily different from any sensible reconstruction of an expressivist position. The expressivist is committed to the organic model, while MacFarlane’s position illustrates the building-block approach. He is right that it is easy to imagine situations in which ‘Licorice is not tasty’ and ‘I know the taste of licorice first-hand and I do like it’ can be thought at the same time, about the same licorice, without the thinker being irrational, but that only shows that ‘Licorice is tasty’ and ‘I know the taste of licorice first-hand and I do like it’ are not analytically equivalent. Whenever real analytically equivalent cases can be found within the organic model, as in 3a to 4c, it is irrational to attribute one but not the other.

The two models can be discriminated by the analytic-equivalence test. The negative answer to the question whether analytically equivalent sentences always express the same proposition characterizes the building-block model of content individuation. The positive answer is the semantic core of the organic model. In the dispute between McFarlane and Gibbard, there is an essential mismatch that underlies their local disagreement about the identification of normative contents with expressions of mental states, which classifies either view under a different model. McFarlane is a representative of the building-block model, while Gibbard represents the organic model. Their views are thus more dissimilar than what meets the eye. Both models have strengths and weaknesses and at the level of first-order contents the two parties propose possibly compatible accounts. Nevertheless, when functions of propositions are involved, the analytic-equivalence test settles the issue for the organic model. Only the organic model agrees with the speakers’ intuitions and thus it is the only one appropriate for the analysis of higher-order functions, in general, and functions of propositions, in particular. We might reject that the speakers’ intuition plays any role in the analysis of meaning, as the proponents of the various error theories do, but this move would take the study of language away from the game of science. We chose the empirical path in ‘Minimal Expressivism’ (Frápolli and Villanueva, 2012) by assuming that semantic hypotheses on the behavior of functions of propositions were, in this sense, *a posteriori*. The analytic-equivalence test adjudicates between the principle of compositionality and the principle of propositional priority and confirms that when higher-order concepts are at stake, expressivism is the correct approach.

## Conflict of Interest Statement

The authors declare that the research was conducted in the absence of any commercial or financial relationships that could be construed as a potential conflict of interest.

## Footnotes

1 “What I’m advocating for normative terms is very different from contextual relativism, so different that in my 1994 paper I decided not to call it ‘relativism’ at all, and to label it a kind of expressivism (though one very different from old-fashioned versions of expressivism, in that it gives evaluative statements a cognitive role). But MacFarlane (2007) has recently introduced the term ‘assessor-relativism’ for what seems at first blush to be just this sort of thing” (Field, 2009, p. 252).

2 An argument could be made to the effect that Relevance Theory does actually belong to the theories grouped under the “organic model” label. Within Relevance Theory, individuation of content is performed with the aid of the presumption of optimal relevance – the cognitive impact of an utterance needs to match the effort that is required to interpret it. As the cognitive impact of an utterance is established with respect to the status of the audience’s belief box at the time of the utterance, whether the presumption of optimal relevance is upheld can only be determined by paying attention to the whole judgment, instead of its sub-sentential components. Moreover, Relevance Theory acknowledges the existence of top–down pragmatic processes, even acting from the level of implicatures, with an impact on the explicature. These reasons, the presumption of optimal relevance as a guiding principle for content individuation and the existence of top–down pragmatic processes, could be sufficient to persuade some of the idea that Relevance Theory is unfairly listed within the building-block model theories. Tempting as this might be, we think that this inclination must be resisted, for the following reason: the sheer distinction between bottom–up and top–down pragmatic processes *only makes sense* within a building-block background. Relevance Theory’s commitment with a logical form that gets enriched with different components was essential to the position as it was introduced, and continues to be part of the standard description of the theory (Carston, 2000, p. 10; Clark, 2013, p. 305; Romero and Soria, 2014, p. 490).

3 “The less I have said about what so-called semantic values must be, the more I am entitled to insist on what I did say. If they don’t obey the compositional principle, they are not what I call semantic values” (Lewis, 1980, p. 91).

4 Reverse Compositionality (Fodor, 1998, cfr. Szabó, 2013) is no better candidate to do justice to Frege’s ideas on this issue. If the principle is interpreted as a sort semantic version of “reverse engineering,” then it is incompatible with the Fregean stance regarding the fact that multiple logical forms can result from the analysis of a single judgment. If it only amounts to the platitude that whatever the analysis of a judgment, the final components should be somehow related to the whole, then it is both compatible with the organic and the building-block models. Similarly, if it is only meant as ‘a statistical psychological generalization that holds with great regularity’ (Johnson, 2006, p. 52), then Reverse Compositionality is not particularly useful when discussing content individuation.

5 It might perhaps be surprising for some not to find a mention in this section of Donald Davidson, one of the best-known champions of the cause against the organic model. The reason for this is that we wanted to avoid any possible confusion between holism, Davidson’s own brand of anti-building-block theory, and expressivism, which is the target of this paper. The kind of contemporary expressivism that we explore in this paper is different from holism –and from ‘global expressivism’ (Price, 2011), or inferentialism– at least in two crucial aspects: expressivism is not committed to the idea that *every* expression needs to receive an organic analysis, and expressivism does not need to accept that every inference is a *meaning-determining* inference (cfr. Gibbard, 2012, p. 109 and ff.).

6 Please note that our claims concerning expressivism and relativism, but also the building-block model and the organic model, concern only the *individuation of content*. Thus we take them to be for the most part orthogonal with respect to the much debated issue of the identity of propositions. Our goal is to explore when two contents differ, rather than to establish what propositions are.
